# Supplementary material for: Teichoic acids anchor distinct cell wall lamellae in an apically growing bacterium
Source: Commun Biol. 2020 Jun 17;3:314. doi: 10.1038/s42003-020-1038-6 (PMC7300013; doi:10.1038/s42003-020-1038-6)
Supplement: Supplementary file 1 — Supplemental Information [file 42003_2020_1038_MOESM1_ESM.pdf]

Teichoic acids anchor distinct cell wall lamellae in an apically growing bacterium

SUPPLEMENTARY INFORMATION

Supplementary figures

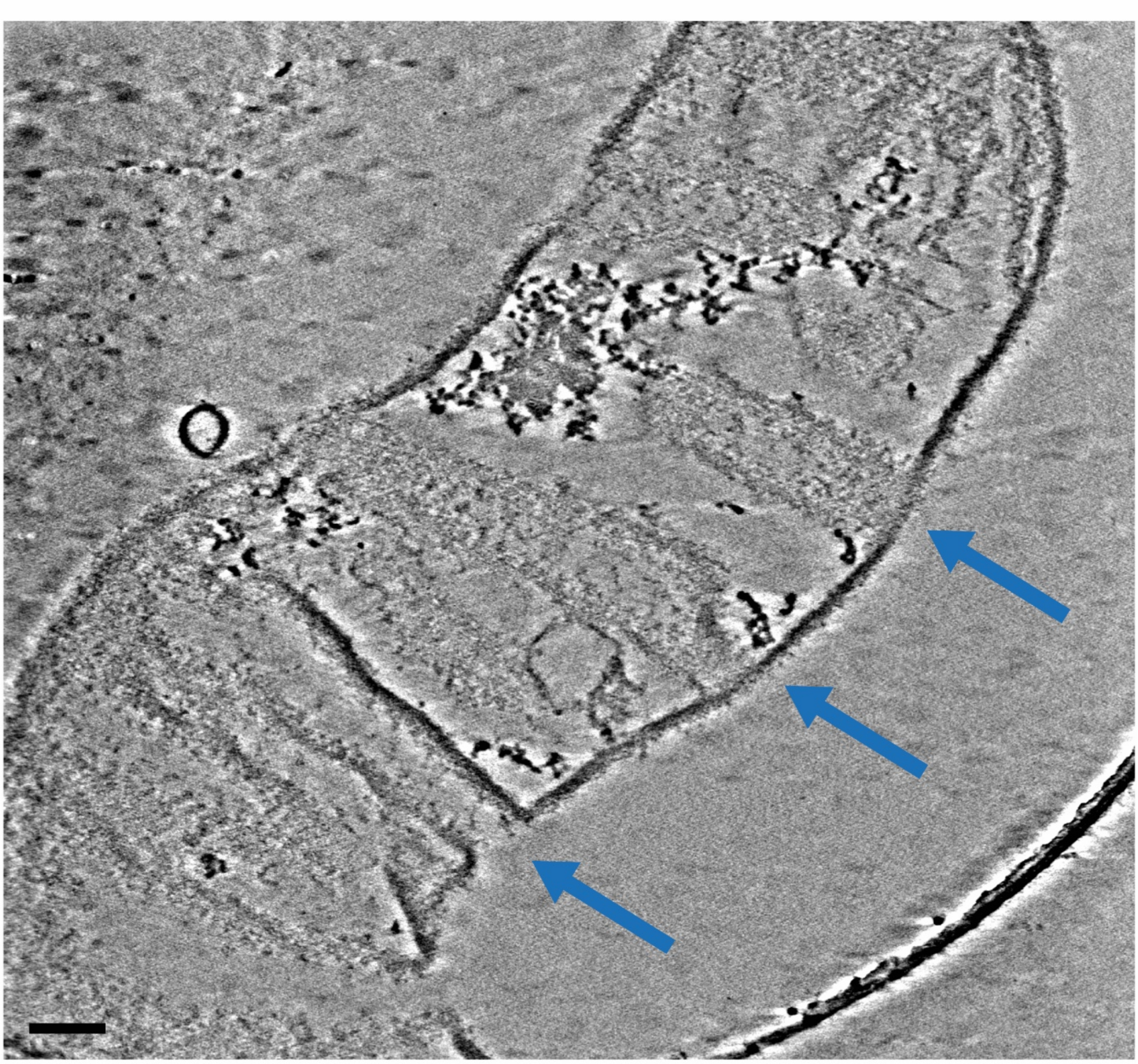

Supplementary Figure 1. Cryo-electron tomograms of isolated *S. coelicolor* sacculus

Example of a cryo-electron tomogram of an *S. coelicolor* sacculus showing multiple folds perpendicular to the cellular axis. Folds are highlighted by the blue arrows. Scale bar 100 nm.

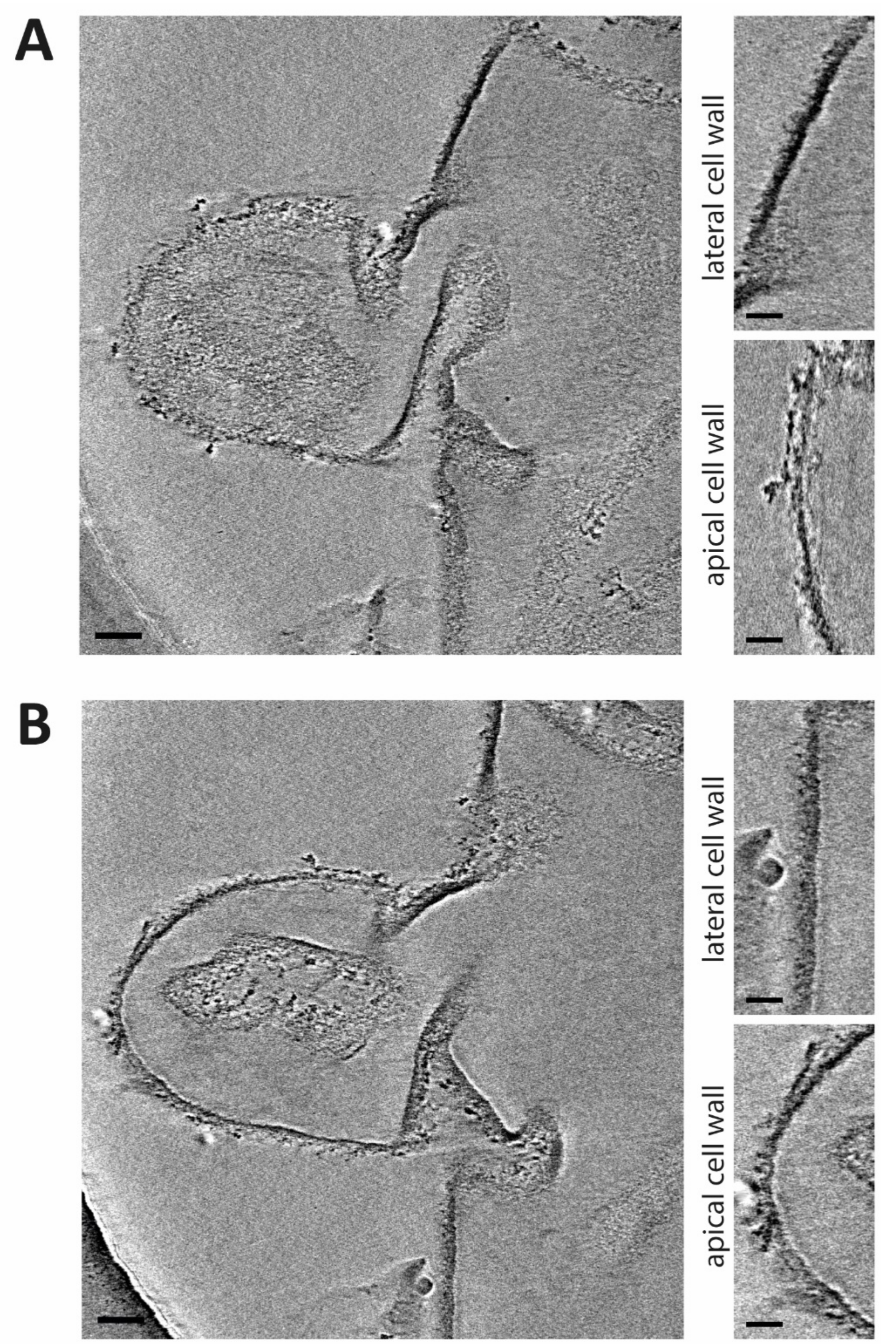

Supplementary Figure 2. Cryo-ETs of HF-treated *S. coelicolor* sacculus with a side-branch

Cryo-ET micrograph of a side-branch. The two large panels A and B are micrographs taken from two different Z heights to accurately compare the cell wall morphology of the folded sacculus. The insets on the right side are from the lateral sides of the hyphae and of the apical cell wall at the tip of the newly formed branch. Scale bars 100 nm, insets 20 nm.

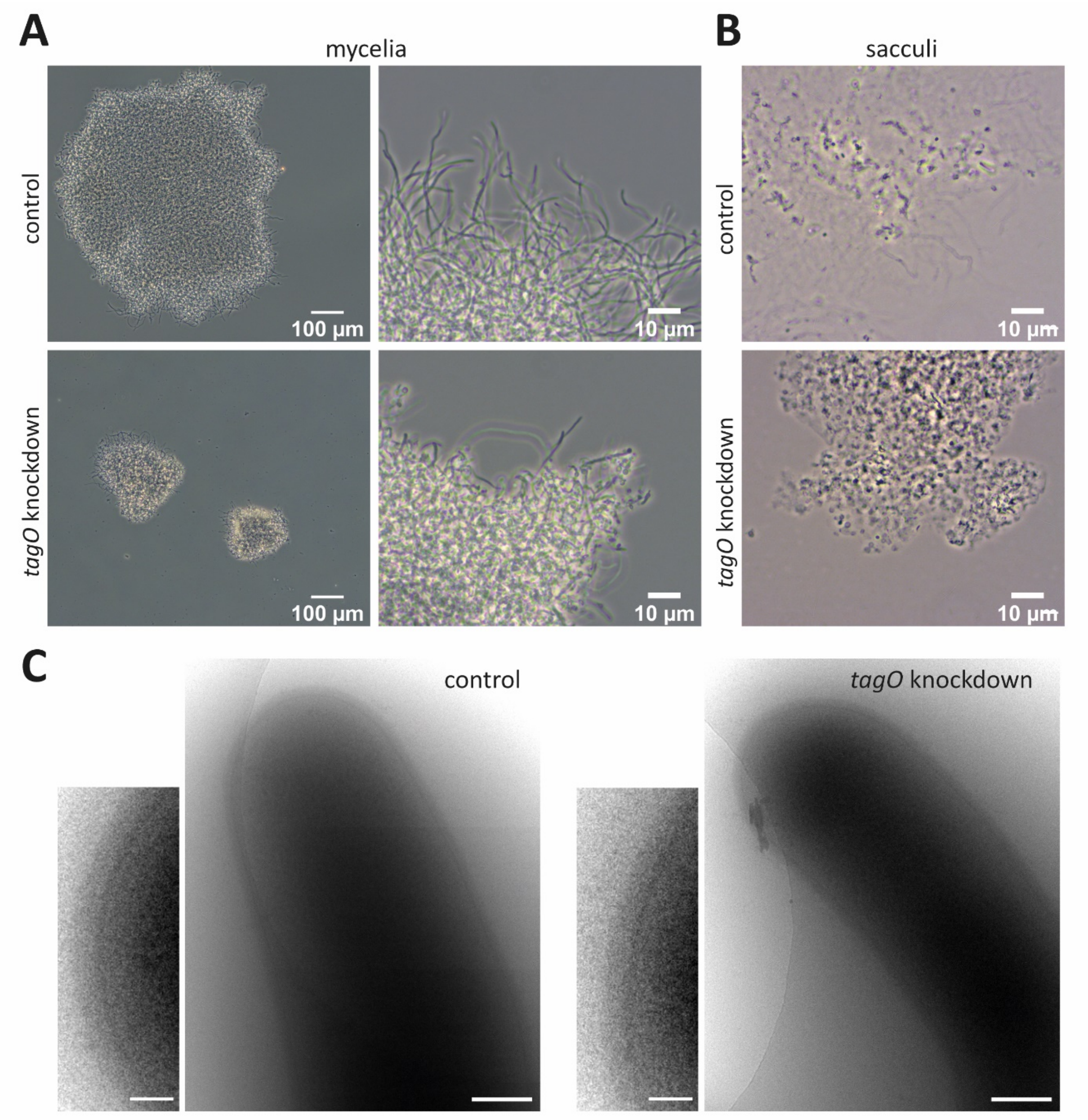

Supplementary Figure 3. ***S. coelicolor tagO*** **knock-down shows no morphological difference in the cell envelope**

The *S. coelicolor* M145 *tagO* knockdown strain and control strain were cultured in TSBS liquid broth for 36 hours, after which the sacculi were isolated. The *tagO* knockdown strain showed impaired germination and delayed growth, resulting in clumps of cells and spores upon sacculi isolation. Cryo-TEM of mycelia shows no morphological difference in the cell envelope at the hyphal tip for both strains (B). Scale bars 100 µm and 10 µm (A), 10 µm (B), and 50 nm (insets C) and 200 nm (C).

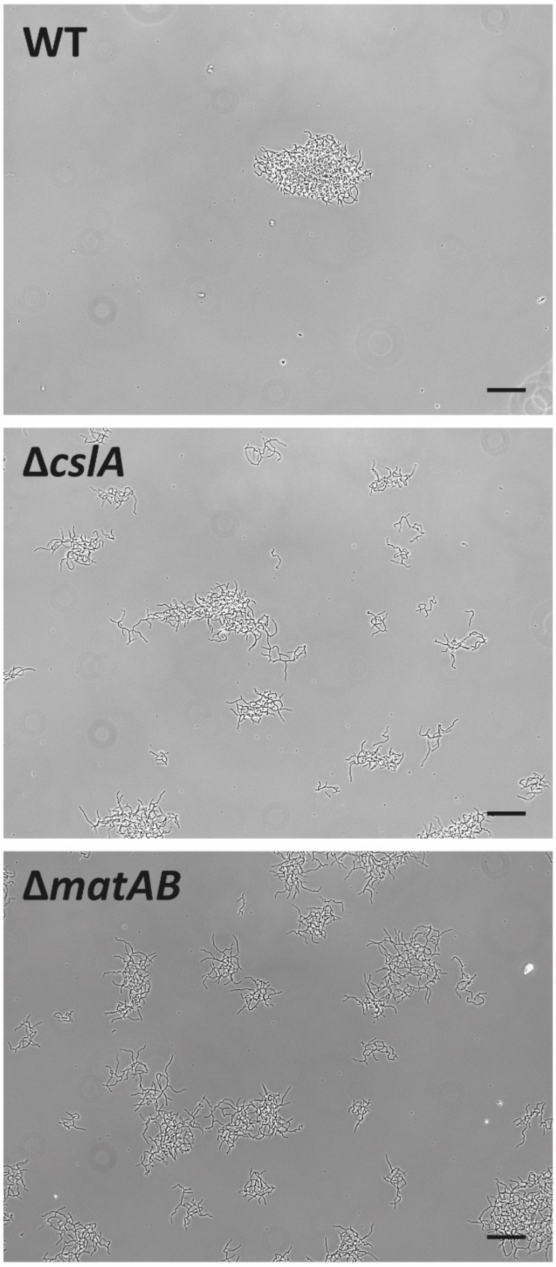

Supplementary Figure 4. HF-treated *S. coelicolor* sacculi degrade upon mutanolysin treatment

The HF-treated *S. coelicolor* sacculi were incubated with mutanolysin for 0, 15 and 30 minutes. The two rows in panel A show light microscopy micrographs, whereas panel B shows room temperature EM to visualize the degradation of the sacculi upon mutanolysin treatment. Scale bars 50 µm and 10 µm (A) and 2 µm (B).

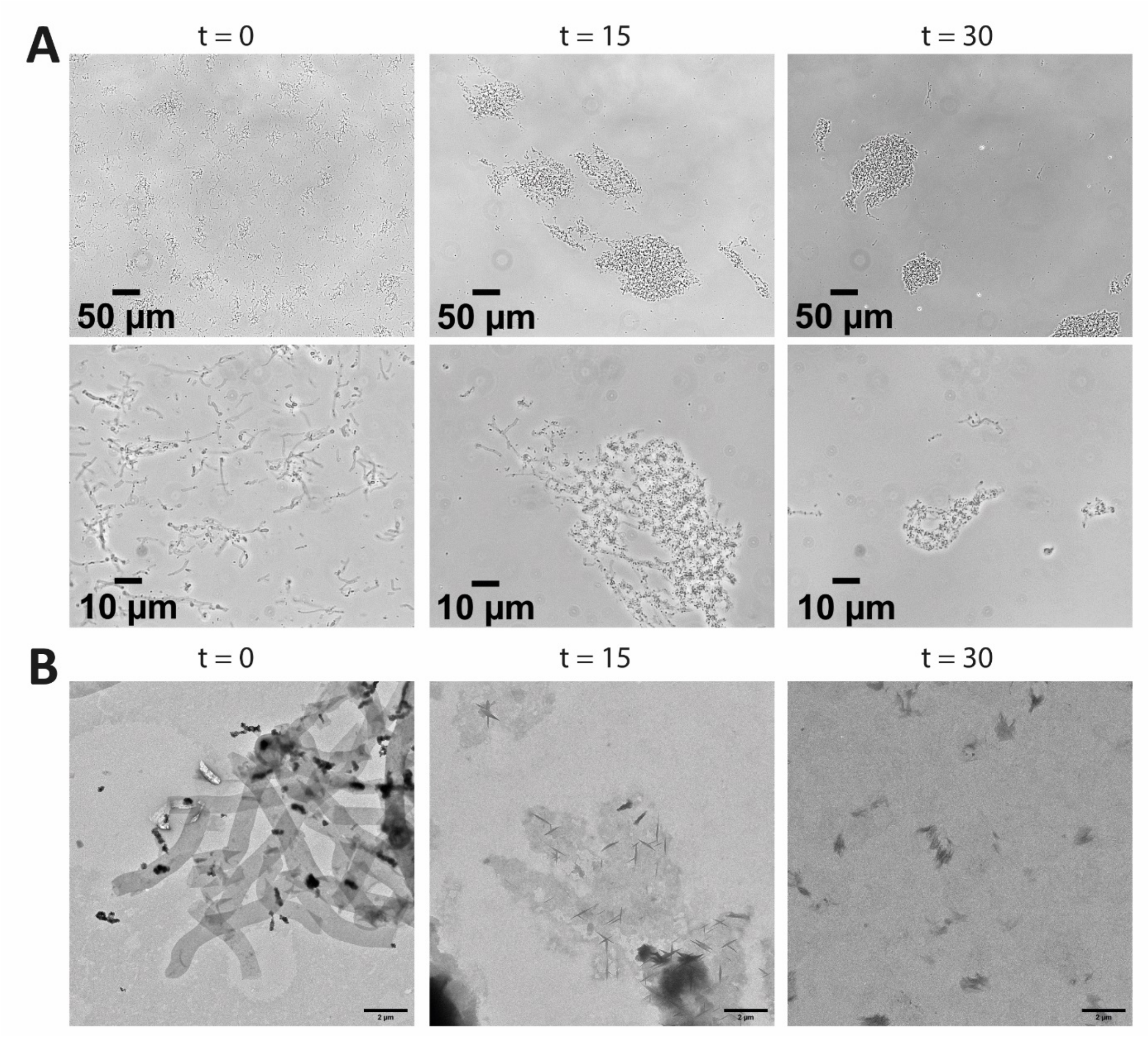

Supplementary Figure 5. Pellet morphology of *S. coelicolor* WT, Δ*cslA* and Δ*matAB*

Pellet morphology of *S. coelicolor* WT, Δ*cslA* and Δ*matAB* after 12 hours of growth in liquid TSBS medium. The WT forms a dense pellet, whereas Δ*cslA* and Δ*matAB* show a dispersed morphology. Scale bars 50 µm.

Supplementary tables

Supplementary Table 1. Overview of plasmids, constructs and oligonucleotides used to create *tagO* knockdown via CRISPRi

Plasmids and Constructs

Plasmid and constructs	Description	Reference
pHJL401	*E. coli/Streptomyces* shuttle vector, around 10-20 per chromosome copies in *Streptomyces* and around 100 copies per chromosome in *E. coli*	[67]
pSET152	*E. coli/Streptomyces* shuttle vector, high copy number in *E. coli* and integrative in *Streptomyces*	[68]
pGWS1045	pHJL401 containing sgRNA scaffold (no spacer)	This work
pGWS1049	pGWS1045 containing *dcas9* under the control of *gapdh* promoter	This work
pGWS1355	pGW1049 containing spacer targeting template strand of *tagO*	This work
pGWS1358	pGWS1049 containing spacer targeting non-template strand of *tagO*	This work
pGWS1362	pSET152 containing sgRNA scaffold with spacer targeting template strand of *tagO* and *dcas9* under the control of *gapdh* promoter	This work
pGWS1365	pSET152 containing sgRNA scaffold with spacer targeting non-template strand of *tagO* and *dcas9* under the control of *gapdh* promoter	This work

Oligonucleotides

Name	5'-3' sequence#
SgPermE_F_EBG	ctag**GAATTC**GAGCTCGGTACCCGGG**AGATCT**ACGCGGTCGATCTT
SgTermi_R_B	ctag**GGATCC**CAAAAAACCCCTCAAGACCCGTTTAGAGGCCCCAAGGGGTTATGC TAGTTACGCCTACGTAAAAAAAGCACCGACTCGGTGCC
Pgapdh_F_(E)B	**catg**GAATTC**GGATCC**gctgctccttcggtcggacgt
Pgapdh_R_(H)NdeI	catgAAGCTT**CATATG**GCGTATCCCCTTTCAGATACTC
Cas9_F+1_(E)NdeI	catgGAATTC**CATATG**GACAAGAAGTACTCCATCGGC
Cas9Termi_R+4107_XH	ctagAAGCTT**TCTAGA**CTCCGACCGCACGGCCGCAACCGGAGTGGTCCGGAAGCG GCCTGCGGAGCGCCTCAGTCGCCGCCGAGCTGGGACA
TagO_T_F	CATG**CCATGG**CGGTGACGTATCTGCTGACAGTTTTAGAGCTAGAAATAGC
TagO_NT3_F	CATG**CCATGG**GGACACCGATCAGCCAGATCGTTTTAGAGCTAGAAATAGC

Restriction sites used for cloning are underlined and in bold. GGATCC, BamHI; AGATCT, BglII; GAATTC, EcoRI; CATATG, NdeI; CCATGG, NcoI; TCTAGA, XbaI. T7 terminator in primer SgTermi_R_B and *aph* terminator in primer Cas9Termi_R+4107_XH are shown in red. The 20 nt target in primers TagO-T_F and TagO_NT3_F are shown in blue.

Supplementary References

67. Larson, J. L. & Hershberger, C. L. The minimal replicon of a Streptomycete plasmid produces an ultrahigh level of plasmid DNA. Plasmid 15, 199–209 (1986).

68. Bierman, M. et al. Plasmid cloning vectors for the conjugal transfer of DNA from Escherichia coli to Streptomyces spp. Gene 116, 43–49 (1992).
