# Supplementary material for: Teichoic acids anchor distinct cell wall lamellae in an apically growing bacterium
Source: Commun Biol. 2020 Jun 17;3:314. doi: 10.1038/s42003-020-1038-6 (PMC7300013; doi:10.1038/s42003-020-1038-6)
Supplement: Supplementary file 2 — Description of Additional Supplementary Files [file 42003_2020_1038_MOESM2_ESM.pdf]

## Description of Additional Supplementary Files

**File Name**: **Supplementary Data 1**
**Description**: Cell wall density measurements on S. coelicolor sacculi (source data of figure 2A and 2B).

**File Name**: **Supplementary Data 2**
**Description**: Cell wall thickness measurements on S. coelicolor sacculi (source data of figure 5B).
